# Network Analysis Highlights Complex Interactions between Pathogen, Host and Commensal Microbiota

**DOI:** 10.1371/journal.pone.0084772

**Published:** 2013-12-23

**Authors:** Sébastien Boutin, Louis Bernatchez, Céline Audet, Nicolas Derôme

**Affiliations:** 1 Institut de Biologie Intégrative et des Systèmes (IBIS), Département de Biologie, Université Laval, Québec, Québec, Canada; 2 Institut des sciences de la mer de Rimouski (ISMER), Université du Québec à Rimouski (UQAR), Rimouski, Québec, Canada.; University of North Carolina at Chapel Hill, United States of America

## Abstract

Interactions between bacteria and their host represent a full continuum from pathogenicity to mutualism. From an evolutionary perspective, host-bacteria relationships are no longer considered a two-component system but rather a complex network. In this study, we focused on the relationship between brook charr (*Salvelinus fontinalis*) and bacterial communities developing on skin mucus. We hypothesized that stressful conditions such as those occurring in aquaculture production induce shifts in the bacterial community of healthy fish, thus allowing pathogens to cause infections. The results showed that fish skin mucus microbiota taxonomical structure is highly specific, its diversity being partly influenced by the surrounding water bacterial community. Two types of taxonomic co-variation patterns emerged across 121 contrasted communities’ samples: one encompassing four genera well known for their probiotic properties, the other harboring five genera mostly associated with pathogen species. The homeostasis of fish bacterial community was extensively disturbed by induction of physiological stress in that both: 1) the abundance of probiotic-like bacteria decreased after stress exposure; and 2) pathogenic bacteria increased following stress exposure. This study provides further insights regarding the role of mutualistic bacteria as a primary host protection barrier.

## Introduction

Ever since their emergence, eukaryotes have lived in close interactions with microorganisms [1]. Those interactions take many forms, from neutralist to closest relationships, and their effects on the host will vary from highly beneficial (mutualism) to detrimental (parasitism, pathogenesis). The complexity of bacterial communities inhabiting fish skin mucus has been rarely studied. However, it is well established that this microbiota share species with the surrounding water and that some opportunistic pathogens may be found on healthy fish [2,3]. Yet, it is not clear whether an asymptomatic pathogen occurrence represents a latent step of a disease cycle, a first colonization prior to pathogenesis, or a commensalism/mutualism interaction. Opportunistic pathogens are present in healthy fish microbiota, and may become infectious when hosts are stressed [4]. As an external organ, fish skin is an important first-line defense system against pathogens [5]. Interactions between fish and bacteria on the skin surface are used as a model in human investigative dermatology [6]. Recent advances in the field of evolutionary and ecological mechanisms governing human symbiotic communities shed a light on the microbiota functions associated to host health. Furthermore, disturbance of such microbial interacting networks are linked to specific human diseases [7]. Alteration in relative abundance of phyla constituting the microbiota is called dysbiosis [8]. Although mechanisms leading to dysbiosis are not yet understood, their outcome weakens the first-line immune function of the mucosal barrier [9]. Fish indigenous bacteria contribute to the immune function of the skin mucus with several mechanisms: colonization resistance (CR) which is preventing pathogen growth with competitive use of resources [8], friction preventing polymers, immune response stimulation or production of inhibitory compounds [2,10]. The decrease of commensal bacteria expressing those functions in fish skin mucus is expected to allow increased abundance of potentially pathogenic organisms.

In order to explore the complexity of host-bacteria interactions, experimental methods with high resolution are desperately needed. Despite their usefulness, DGGE, cloning, or culture based-methods have shown limitations in accurately characterizing complex bacterial communities and rare biosphere [11–15]. The emergence of novel high-throughput sequencing technologies provides high-definition tools to deeply investigate complex taxonomic assemblages in bacterial communities [16,17]. This allows visualizing both the composition (i.e. diversity) and the structure (i.e. relative abundance of species) of bacterial communities. Also, because bacterial species rather exist as complex and interactive networks than as monospecific colonies [18–20], network analysis represents a promising avenue towards investigating the co-occurrence and taxonomic interactions among species in complex bacterial communities [16,19]. 

In this study, we characterized the relationship between brook charr (*Salvelinus fontinalis*) and its skin-associated microbiota in order to test the general hypothesis that stressful conditions exerted on healthy fish, as those occurring in aquaculture production, induce a shift in the microbiota taxonomic structure, which in turn potentially triggers opportunistic pathogen infections. To this end, we aimed three specific objectives; i) to study the effect of the different factors on fish skin microbial community, including: environmental bacterial communities, host genetic background and host physiological stress, ii) to deeply characterize the endogenous skin microbiota of brook charr (*Salvelinus fontinalis*) and its relationship with the biodiversity occurring in the surrounding environment bacterial community, and iii) to understand the dynamics of those communities using co-occurrence network analysis.

## Materials and Methods

### Ethics statement

All fish were reared and the experiment conducted strictly following guidelines required by the ‘‘Comité de Protection des Animaux de l’Université Laval (CPAUL, http://www.vrr.ulaval.ca/deontologie/cpa/index.html?accueil). The CPAUL reviewed and approved all experimental procedures used in this study.

### Fish rearing

Brook charr from the Laval strain were produced and reared at the ISMER (Institut des sciences de la mer de Rimouski) aquaculture laboratory, and fish were transferred at LARSA (Laboratoire Régional des Sciences aquatiques), U. Laval, Québec to conduct the experiments. We used individuals issued from eight different full-sib families (80 individuals by family). Families were identified by removing the apidose and/or pelvic fins [21]. All fish were reared in the same conditions; 10°C, natural photoperiod and were fed daily with commercial pellets according to biomass and temperature. According to the CPAUL guidelines, fish were transferred in two independent rearing units to avoid pathogen outbreaks between test and control tanks. Each unit was composed of four tanks sharing the same biofilter and water circuit. In each tank, two fish families were mixed and the distribution of the families was the same between the test and the control unit to avoid bias. All the parameters of the water and the rearing system were set for the well-being of the fish and to decrease the stress as much as possible. Water was sterilized with a UV lamp before entering into rearing units. 

### Stress experiment

One unit was exposed to an acute stress (high density and hypoxia) for testing the effect of stress on skin microbial communities, and the other unit was used as control. The experiment began after a one-month acclimatization period. To induce stress, we performed an intense hypoxia exposure in the test unit. Fish were transferred in a new tank without the input of oxygen. Then, the fish were exposed to high density (80 fishes in 10 L) until the oxygen concentration decreased to 3 mg/L (5 min). After stress exposure, fish were transferred in a new oxygenated tank to slowly recover. Control fish were moved to another tank to be anesthetized with MS-222 (0.05 g/L) like the stressed fish, but control fish were not subjected to high density and hypoxia. To avoid contamination or the effects of sterile water, the tanks used for stress and recovery were filled with the same water that the initial tanks. After a full recovery, fish and water were returned to their initial tanks.

### Sampling

**Figure 1:**
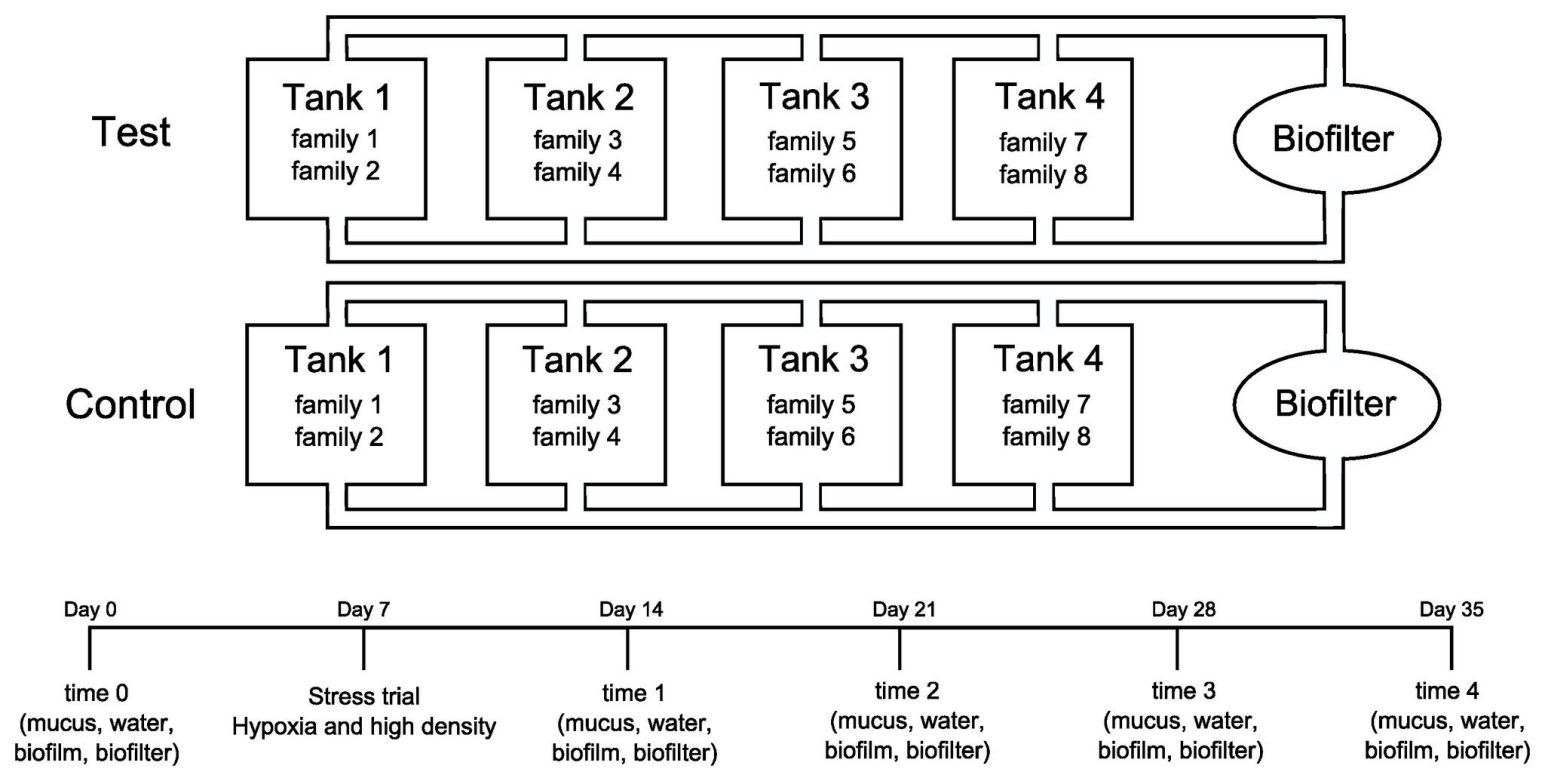
Scheme of the study and sampling design. The design comprise two different units (test vs control) composed of four tanks, all sharing the same water and biofilter. Fish were equally split between the two units and were raised in the same conditions. Only the fish from the test unit were exposed to stress. The sampling design is a 35 days protocol. On day 0, we sampled the time 0 bacterial communities from mucus, biofilters, biofilm and water. On day 7, the fish from the test unit were exposed to the stress. On day 14, 21, 28 and 35, we sampled the bacterial communities (samples time 1, 2, 3 and 4 respectively) from mucus, biofilters, biofilm and water.

Ten individuals from each family were randomly sampled at five different time periods during the experiment: 1) before the stress trial (time 0), 2) stress trial at one week, 3) one week (time 1), 4) two weeks (time 2), and 5) three weeks (time 3), and 4 weeks after exposure to stress (time 4) (Figure 1). When death occurred, samples were also taken on these animals the day they died. For all samplings, fish were anesthetized with MS-222 (0.05 g/L) and family was identified. Baseline of stress response was determined by sampling blood one week before the stress experiment. Blood samples were taken by caudal puncture using a sterile syringe. The same procedure was done on ten other individuals from each family, 10 minutes after the stress experiment to measure the response to stress. Quantification of plasma cortisol concentration was used as a proxy of stress status [22–24]. Plasma cortisol was measured by radioimmunoassay with an Immuchem-coated tube kit (MP Biomedical, LLC, NY). Each family’s samples were pooled together (ten individuals) and duplicate measurements were done on each sample. For each assay, the plasma concentrations of cortisol were determined following a standard curve constructed with results obtained from cortisol standards provided in the kit. Mucus samples were taken with sterile swabs on the surface of the fish [25]. Samples were put into sterile micro-centrifuge tubes containing lysis buffer (Tris 50 mM, EDTA 40 mM, Sucrose 0.75 g) and stored in a - 80°C freezer until DNA extraction. 

For water bacterial community sampling, a two-step filtration was performed using peristaltic filtration equipment (Masterflex L/S Pump System with Easy-Load II Pump Head, Cole-Parmer) cleaned up with HCl 5% and rinsed with Milli-Q water before each filtration. The first step is the conditioning of the filtration set up using two liters of the tank rearing water without filters. The last step was to collect duplicates of water from test and control units, by filtering a total of four liters of tank water over a series of filters beginning with a 3.0 μm followed by a 0.22 μm x 47 mm nitrocellulose membrane (Advantec). Immediately, after each filtration process, filters were placed into cryotubes containing 1 mL of sterile lysis buffer (40 mM EDTA, 50 mM Tris-HCl, 0. 75 mM sucrose) and then stored at -80°C until DNA extraction.

For biofilm sampling, water level on each unit was lowered and biofilm was sampled at each time-point with a sterile swab on the edge of the tank. Samples were put into sterile micro-centrifuge tubes containing lysis buffer (Tris 50 mM, EDTA 40 mM, Sucrose 0.75 g) and stored at -80°C until DNA extraction. For biofilter sampling, a piece of styrofoam peanut was taken from the biofilter, placed into a sterile micro-centrifuge tube containing lysis buffer (Tris 50 mM, EDTA 40 mM, Sucrose 0.75 g), and stored in a -80°C freezer until DNA extraction.

### DNA extraction and cortisol measurements

DNA was extracted from the four different types of samples (mucus, water, biofilter and biofilm) using a modified salt-extraction protocol [26]. During the first lysis step, 22.6 µL of lysozyme (40 mg/mL) was added to the sample and incubated 45 minutes at 37°C. After this step, 22.6 µL of proteinase K (20 mg/µL) and 90 µL of SDS (10%) were added to the lysate and incubated at 55°C over night with agitation. All the aqueous phase was transferred into a clean Eppendorf tube containing 600 µL of NaCl 6M, mixed and centrifuged 20 min at 14 800 rpm. The supernatant was transferred again into a clean Eppendorf tube containing one volume of cold isopropanol, mixed, and stored 30 minutes at -20°C. The mixture was centrifuged 20 minutes at 14 800 rpm, and the supernatant was thrown away. The pellet was washed with cold ethanol 70%, air dried, and finally resuspended in 25 µL of sterile Milli-Q H_2_O. Subsequently, DNA integrity and quantity was controlled using a Nanodrop instrument (ND-1000, Nanodrop). 

### Massive parallel pyrosequencing

For each DNA sample, the 16S rRNA gene was PCR amplified using Takara Ex Taq premix (Fisher). All PCR reactions were performed in a final volume of 50 μL containing 25 μL of Premix Taq, 1 μM of each primer and sterile Milli-Q H_2_O to reach 50 μL. To achieve the PCR amplifications, a general reverse primer (R519) combined with B primer (Roche) was used in combination with a unique tagged forward primer (F63-targeted) combined with A primer (Roche) (for primer sequences see [27,28]). These primers were chosen to target the hypervariable region 1 to 3 of the 16S rRNA gene [29]. PCR conditions were as follow: after a denaturing step of 30 s at 98°C, samples were processed through 30 cycles consisting of 10 sec at 98°C, 30 sec at 55°C, and 30 sec at 72°C. The final extension step was done at 72°C for 4 min 30 sec. Following amplification, samples were purified using AMPure Beads (Beckman Coulter Genomics). Samples were adjusted to 100 µL with EB (Qiagen), 63 µL of Beads was added. Samples were mixed and incubated for 5 min at RT. Using a Magnetic Particle Concentrator (MPC), the beads were pelleted against the wall of the tube, and supernatant was removed. The beads were washed twice with 500 µL of 70% ethanol and incubated for 30 sec each time. Supernatant was removed, and beads were allowed to air dry for 5 min. Tubes were removed from the MPC and 24 µL of EB was added. Samples were vortexed to resuspend the beads. Finally, using the MPC, the beads were pelleted against the wall once more and the supernatant was transferred to a new clean tube. Samples were quantified with Nanodrop and mixed equally before being sent to the Plateforme d’Analyses Biomoléculaires (Institut de Biologie Intégrative et des Systèmes, Université Laval) for sequencing on a 454 GS-FLX DNA Sequencer with the Titanium Chemistry (Roche), according to the procedure described by the manufacturer. 

### Sequence analysis

All sequences are available on MG-RAST server (MG-RAST IDs: 4536758.3, 4536759.3). The data were analyzed in two steps. First, CLC Genomics Workbench 3.1 (CLC Bio, Aarhus, Denmark CLC workbench BIO®) was used to trim sequences for quality and recover the primers' sequences and tags (minimum average quality score: 35 for a window of 50, number of differences to the primer sequence = 0, maximum number of differences to the barcode sequence = 0, number of ambiguous base calls = 0, maximum homopolymer length = 8). In a second step, pre-processing and analysis were performed using the MOTHUR software v.1.29 [30]. All datasets were checked for chimeras with the chimera slayer algorithm implemented in MOTHUR. Standardization of the different samples was done by using the zscore which calculates the normalized abundance: Normalized Abundance = (Abundance - mean) / standard deviation. We used the Operational Taxonomic Unit-based method [31] because it is not biased by a predefined taxonomy. We estimated the alpha-diversity by calculating the non-parametric index of Shannon. The Good’s coverage index was retained to assess the quality of the sampling effort [32]. Beta-diversity between the different sample categories (mucus, water, biofilm and biofilter) was explored by constructing a phylogenetic tree based on a phylip-formatted distance matrix containing all the 16S sequences. Statistical robustness of the tree was determined by a Unifrac weighted test because it allows determining whether any of the samples have a significantly different structure than the other groups. A random (Monte Carlo) permutation test was performed to test whether or not the distance between two communities was greater than expected by chance alone. To visualize those taxonomical relationships between mucus and water communities, distances between communities were computed using the Yue & Clayton measure of dissimilarity (Thetayc) and then represented using a dendrogram based on Thetayc (weighted) indices [33]. All sequences were clustered into OTU using a 97% identity threshold and OTU were classified from phylum to genus using the program MOTHUR with the default setting. To highlight the influence of the different environmental factors (family, stress, tank) on the community structure, an ANOVA was performed on a generalized linear model (family:quasibinomial).

To identify potential taxonomic interactions between genera, we analyzed the non-random co-occurrence for each genus. The first step was to measure the co-occurrence value, which was then compared to that was expected by chance. We used the ecological measure based on the checker-board unit C-score [34]. Bacterial networks were built by first computing all possible Spearman’s rank correlations between genera with more than 10 sequences in the whole dataset (151 genera). We considered a valid co-occurrence event to be a robust correlation if the Spearman’s correlation coefficient was both > 0.6 and statistically significant with Bonferroni correction (P-value < 0.01) [19]. Network was constructed using Fruchterman-Reingold layout in Igraph package (iterations = 500, maximum change = 45, area parameter = 2025, cooling exponent = 3, cancellation radius = 91125) [35]. The nodes of the reconstructed network represent the genera, whereas the edges (i.e. connections) correspond to a strong and significant correlation between nodes. All statistical analyses and graphics were carried out in the R environment with the package multtest, Hmisc, Foreach and Igraph (http://www.r-project.org).

## Results

A total of 678 211 reads were obtained from the 121 samples, and 468 232 reads were kept after the filtering process of short and bad quality sequences. All of these sequences were successfully clustered in OTU with 97% identity, and were assigned to 16 904 genus distributed among 21 phyla. In order to focus on the most abundant taxa, OTU represented by less than ten reads were discarded. The resulting data set encompassed 376 199 sequences distributed among 390 OTU, 151 genera and 10 groups or phyla. The mean number of reads for the 121 samples was 3109, and the Good’s coverage estimations ranked between 70% and 99% (Table S1).

### Factors influencing fish skin microbiota structure

The analysis of variance (ANOVA) for each genus revealed that the three factors tested (stress, family, and tank) had a significant influence on the structure of the skin fish microbiome (abundance of the genera). The most ubiquitous factor was the stress trial, which explained the variation of 115 genera (76%) and was the most influential factor on the abundance of 78 genera (51%) (Table 1; Table 2). The second most influential factor was the family genetic background which explains the variation of 88 genera (58%) and was the most important factor for 31 genera (20%) (Table 3). Finally, the least widely influent factor was the localization in the tank which is involved in the variation of 48 genera (31%) and is the most influent factor for 18 genera (12%) (Table 4). 

**Table 1:** ANOVA of the influence of the different independent variables (family, stress, tank) on the relative abundances of genera.

**Genus**	**Stress**	**Family**	**Tank localization**	**Mean abundance**
**Alkanibacter**	0.0003817	0.0067909	N.S.	0.00012452
**Arthrobacter**	1.36E-03	0.005906	N.S.	0.00057873
**Azomonas**	0.009495	N.S.	N.S.	0.00585365
**Brevundimonas**	5.85E-05	0.0037	0.002729	0.00626585
**Corynebacterium**	0.002941	N.S.	N.S.	0.00096444
**Duganella**	8.60E-05	N.S.	0.00754	0.00197915
**Elioraea**	0.0001565	0.0009162	N.S.	0.00037959
**Erythromicrobium**	0.0001913	N.S.	N.S.	0.00369634
**Flavobacterium**	0.003698	0.007852	N.S.	0.01814291
**Haematobacter**	0.0002524	N.S.	N.S.	8.3449E-05
**Hydrocarboniphaga**	4.76E-05	N.S.	N.S.	0.00057531
**Hyphomicrobium**	2.68E-05	0.002044	0.00835	0.00040407
**Kocuria**	1.41E-05	0.000329	0.006223	0.00016603
**Kordia**	1.40E-05	0.0001154	0.0061891	0.00012529
**Kordiimonas**	0.0006209	0.0062936	N.S.	0.00065794
**Lysobacter**	8.68E-05	N.S.	N.S.	0.00036283
**Martelella**	0.000359	N.S.	N.S.	0.00029309
**Methylibium**	0.002786	0.003027	N.S.	0.00069505
**Methylobacterium**	0.0001642	N.S.	0.0020754	0.0300864
**Methylosoma**	3.09E-05	0.003439	0.008925	0.00014531
**Mycobacterium**	0.001914	N.S.	N.S.	0.00488646
**Nannocystis**	0.0003806	0.0011184	N.S.	3.0826E-05
**Nesterenkonia**	1.17E-06	7.82E-05	0.001916	0.00026393
**Nevskia**	8.81E-06	N.S.	0.00403	0.00598778
**Nitrosomonas**	0.0001569	N.S.	N.S.	9.9833E-05
**Nitrosospira**	0.0003808	0.0073847	N.S.	5.4431E-05
**Novispirillum**	0.006496	N.S.	N.S.	0.00062797
**Novosphingobium**	1.15E-06	N.S.	0.002174	0.00651896
**Oceanibulbus**	< 2e-16	< 2e-16	< 2e-16	4.3303E-05
**Pseudoxanthomonas**	4.27E-07	7.96E-05	0.001324	0.00105699
**Ralstonia**	1.81E-05	1.69E-06	0.006982	6.0433E-05
**Rhodoferax**	3.25E-07	N.S.	0.011	0.00187594
**Thermodesulfobium**	1.48E-06	N.S.	0.0005346	5.1126E-05
**Thiorhodospira**	0.0002937	N.S.	N.S.	6.8315E-05
**Vulcanithermus**	0.0002274	N.S.	N.S.	6.2458E-05

The table lists the genera, which are mostly influenced negatively by the factor stress. The values correspond to the q-values of the ANOVA (FDR-correction).

**Table 2:** ANOVA of the influence of the different independent variables (family, stress, tank) on the relative abundances of genera.

**Genus**	**Stress**	**Family**	**Tank localization**	**Mean abundance**
**Acinetobacter**	3.59E-07	8.00E-07	N.S.	0.00289109
**Aeromonas**	6.97E-12	3.51E-05	0.0292	0.00020244
**Ahrensia**	7.24E-09	0.002071	N.S.	4.663E-05
**Alkanindiges**	2.68E-07	0.0001153	N.S.	1.8286E-05
**Anaeromyxobacter**	5.19E-11	3.09E-05	N.S.	0.00015175
**Arenimonas**	3.35E-08	1.09E-05	0.03959	0.00013987
**Bacteroides**	6.54E-08	0.0005315	N.S.	5.3673E-05
**Bradyrhizobium**	0.0004757	N.S.	N.S.	0.00245418
**Dechloromonas**	2.69E-07	1.15E-05	N.S.	4.287E-05
**Enterobacter**	0.0002872	N.S.	N.S.	0.00509315
**Escherichia_Shigella**	0.005385	N.S.	N.S.	0.00198075
**Exiguobacterium**	2.94E-08	5.45E-05	N.S.	2.1063E-05
**Filimonas**	2.68E-07	N.S.	N.S.	2.734E-05
**Haliscomenobacter**	4.68E-06	N.S.	N.S.	7.3809E-05
**Ilumatobacter**	7.85E-10	N.S.	N.S.	0.00192789
**Intrasporangium**	1.21E-08	0.0008099	N.S.	8.6399E-05
**Kerstersia**	<2e-16	N.S.	N.S.	0.00024127
**Kiloniella**	0.0003976	N.S.	N.S.	0.00047771
**Leminorella**	2.09E-07	2.29E-05	N.S.	0.0005117
**Microbacterium**	2.10E-07	N.S.	0.001766	0.00018045
**Microterricola**	1.47E-08	0.0004919	N.S.	0.00040735
**Oceanibaculum**	2.70E-07	N.S.	N.S.	0.00017998
**Paludibacter**	6.69E-09	N.S.	N.S.	0.00033504
**Pedobacter**	2.68E-07	4.60E-05	N.S.	2.3559E-05
**Phascolarctobacterium**	4.99E-09	9.48E-05	N.S.	4.5277E-05
**Piscirickettsia**	2.70E-07	0.002278	N.S.	0.00013337
**Pseudomonas**	0.0001303	N.S.	N.S.	0.00053216
**Pseudonocardia**	3.15E-10	N.S.	N.S.	0.00024579
**Psychrobacter**	4.35E-11	N.S.	0.009452	0.00047063
**Raoultella**	7.25E-08	0.0002328	N.S.	3.211E-05
**Rhodobacter**	1.92E-07	0.001448	N.S.	7.7003E-05
**Rhodococcus**	6.42E-07	0.0001424	0.0001378	0.00029521
**Runella**	2.68E-07	2.68E-07	N.S.	1.4832E-05
**Schwartzia**	2.68E-07	4.09E-05	N.S.	5.4355E-06
**Sphingobacterium**	1.85E-05	2.14E-05	0.005455	8.1535E-05
**Sphingobium**	4.36E-14	3.33E-06	N.S.	0.00017902
**Staphylococcus**	5.97E-08	0.0002155	N.S.	7.3179E-05
**Steroidobacter**	2.74E-11	N.S.	0.01424	0.00040867
**TM7.unclassified**	2.44E-08	3.97E-05	N.S.	0.0001057
**Uruburuella**	1.29E-09	N.S.	N.S.	5.5746E-05
**Variovorax**	2.04E-09	4.68E-09	N.S.	1.1341E-05
**Yersinia**	2.68E-07	N.S.	N.S.	7.0209E-05
**Zhangella**	5.57E-08	1.78E-05	N.S.	3.3173E-05

The table lists the genera, which are mostly positively influenced by the factor stress. The values correspond to the q-values of the ANOVA (FDR-correction).

**Table 3:** ANOVA of the influence of the different independent variables (family, stress, tank) on the relative abundances of genera.

**Genus**	**Stress**	**Family**	**Tank localization**	**Mean abundance**
**Arcobacter**	2.68E-07	1.06E-08	N.S.	7.7125E-07
**Citreimonas**	3.16E-11	6.56E-12	N.S.	6.58135E-06
**Sorangium**	2.68E-07	1.06E-08	N.S.	7.24113E-06
**Pelomonas**	2.68E-07	9.58E-08	N.S.	8.0777E-06
**Blastobacter**	4.13E-09	1.50E-14	3.01E-06	8.88308E-06
**Pseudaminobacter**	2.68E-07	1.06E-08	N.S.	1.39353E-05
**Leptobacterium**	0.0003805	6.52E-05	N.S.	1.79709E-05
**Levilinea**	2.69E-07	1.82E-07	N.S.	2.54447E-05
**Pelagibaca**	0.0003807	6.46E-05	N.S.	2.93261E-05
**Desulfatiferula**	3.67E-06	7.61E-08	0.0001407	2.98361E-05
**Curvibacter**	N.S.	0.001865	N.S.	5.26952E-05
**Undibacterium**	0.000382	2.00E-06	N.S.	5.41701E-05
**acidobacteria_gp6_unclassified**	4.34E-06	2.68E-07	0.003581	7.55431E-05
**Stella**	N.S.	5.46E-05	N.S.	8.50225E-05
**Pseudoclavibacter**	0.0003845	6.42E-07	N.S.	0.000121753
**Hyalangium**	0.0002899	4.04E-05	N.S.	0.000140718
**Aminobacter**	N.S.	0.0007089	N.S.	0.00014811
**Janibacter**	N.S.	0.009263	N.S.	0.00016249
**Rothia**	1.19E-05	3.19E-09	0.005725	0.000178962
**Halotalea**	0.009509	0.002751	N.S.	0.000241804
**Leeia**	1.36E-05	2.76E-07	N.S.	0.000266824
**Maricaulis**	0.009314	0.0004662	N.S.	0.000364817
**Blastomonas**	0.0001444	1.30E-07	N.S.	0.000466861
**Archangium**	0.0001372	0.0001326	0.0038099	0.000599636
**Singularimonas**	N.S.	0.0007448	N.S.	0.000655991
**Veillonella**	0.0002544	2.59E-07	N.S.	0.000658774
**Collimonas**	1.58E-05	5.06E-07	0.005651	0.00126634
**Dinoroseobacter**	0.0002444	6.50E-05	N.S.	0.001376595
**Bosea**	0.0005428	0.0002713	0.0008372	0.001390945
**Pseudorhodobacter**	N.S.	0.0002802	N.S.	4.68386E-05
**Salinibacterium**	0.0003824	8.71E-05	N.S.	9.95107E-05

The table lists the genera, which are mostly influenced by the factor family. The values correspond to the q-values of the ANOVA (FDR-correction).

**Table 4:** ANOVA of the influence of the different independent variables (family, stress, tank) on the relative abundances of genera.

**Genus**	**Stress**	**Family**	**Tank localization**	**Mean abundance**
**Acidovorax**	1.78E-05	0.40812	1.60E-06	7.9641E-05
**Blastochloris**	0.0033989	N.S.	0.0009715	0.00017791
**Chitinilyticum**	0.004489	N.S.	3.86E-09	0.00028049
**Chryseobacterium**	0.0008799	5.51E-10	1.38E-05	6.5659E-05
**Citrobacter**	2.72E-05	0.0009962	5.14E-06	4.8467E-05
**Flectobacillus**	N.S.	0.0004603	6.66E-08	1.7876E-05
**Flexithrix**	0.004097	1.48E-08	< 2.2e-16	3.0892E-05
**Mariniflexile**	4.79E-05	0.0001025	< 2.2e-16	4.2423E-05
**Microcella**	2.65E-05	N.S.	5.57E-11	0.32591863
**Natronocella**	1.02E-13	1.88E-13	< 2.2e-16	9.9185E-05
**Neptuniibacter**	N.S.	8.84E-06	1.38E-13	4.8371E-05
**Obesumbacterium**	0.0006779	N.S.	6.17E-06	6.1069E-05
**Paucibacter**	N.S.	3.34E-05	0.002505	0.0006218
**Phenylobacterium**	0.001355	4.74E-05	6.29E-06	7.0047E-06
**Sporocytophaga**	N.S.	0.0002869	4.74E-12	7.1481E-05
**Stenotrophomonas**	N.S.	0.001572	0.001374	0.00037194
**Thiohalocapsa**	N.S.	2.68E-07	0.0005485	1.8653E-05
**Thiohalospira**	4.70E-07	2.84E-16	< 2.2e-16	1.2244E-05

The table lists the genera, which are mostly influenced by the factor tank localization. The values correspond to the q-values of the ANOVA (FDR-correction).

**Figure 2:**
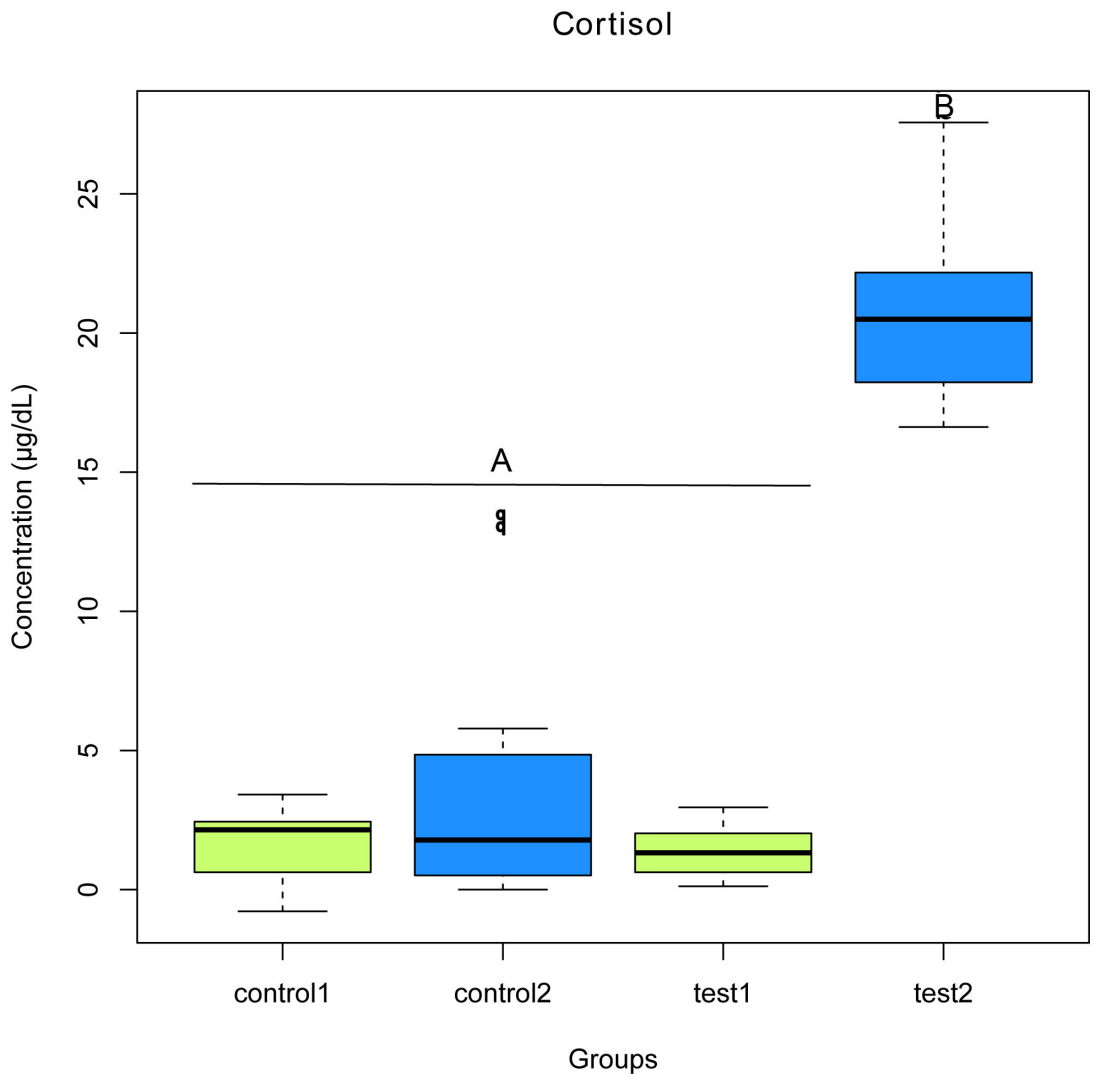
Plasma cortisol concentration in *Salvelinus fontinalis* before and after hypoxia exposure. “Control” represents fish sampled in the control units; “test 1” represent fish sampled before stress exposure in the trial units; “test 2” represents fish sampled 10 min following stress exposure. Each point represents means from 80 fish measurements (10 individuals from 8 families). The presence of statistical significant differences (represented by a and b) was tested by a Wilcoxon rank pair-test (p < 0.001).

The effectiveness of hypoxia and high density exposure as stress inducer was validated through plasma cortisol measurements. Plasma cortisol concentration was low in control fish as well as in the trial units prior to stress exposure. Concentration significantly increased following stress exposure (10 min) (p < 0.001) (Figure 2). Furthermore, a higher mortality rate was observed in the test tanks (45 deaths) compared to control tanks (28 deaths) (p < 0.05). 

### Taxonomic composition of mucus microbiota

**Figure 3:**
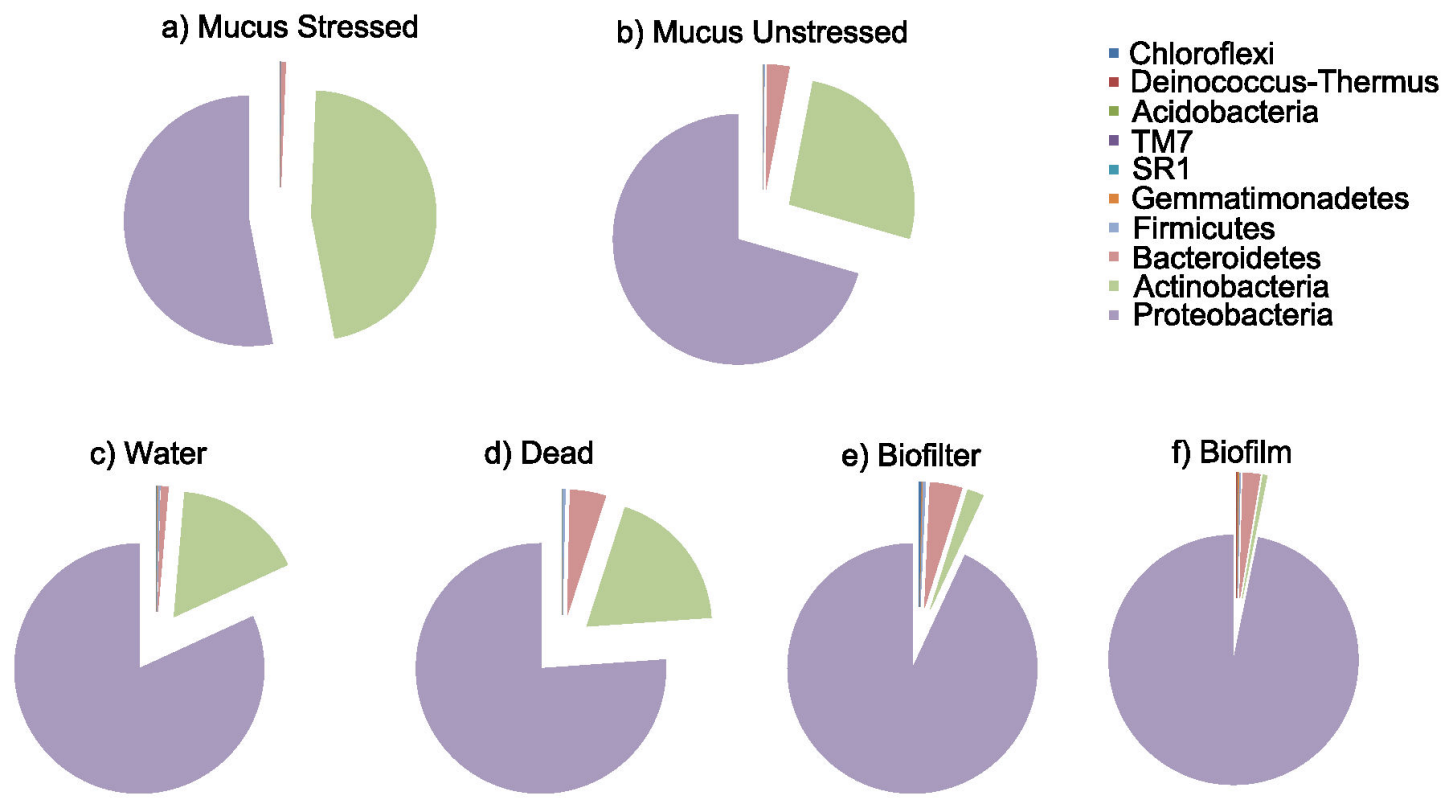
Relative abundance of different microbial taxonomic groups from different communities: a) skin from healthy fish; b) skin from stressed fish; c) skin from dead fish; d) from biofilm; e) from biofilters; f) from water.

All sequences obtained from healthy (48 samples from the control unit and the time 0 sampling of the test unit), stressed (32 samples) and dead fish (11 samples) were classified to genus. The abundant microbial biosphere isolated from fish skin comprised 10 phyla. Three phyla were mostly represented in healthy fish: *Proteobacteria* was the most important (70.6 %), followed by *Actinobacteria* (26.4 %), and *Bacteroidetes* (2.9%) (Figure 3a). At the genus level, the 10 most important genera were *Microcella* (24.4%), *Polynucleobacter* (24.3%), *Sphingomonas* (12.7%), *Thiobacter* (11.9%), *Legionella* (7.9%), *Methylobacterium* (4.2 %), *Flavobacterium* (2.8%), *Novosphingobium* (1.1%), *Brevundimonas* (1.0%), *Nevskia* (0.9%); all of these together accounted for 91.2% of the reads.

In stressed fish, the phylum *Proteobacteria* decreased but remained the most abundant (53%); there was also a lower presence of *Bacteroidetes* (0.52%) (Figure 3b). At the opposite, the occurrence of the phylum *Actinobacteria* increased by two-fold up to 46.3%. At the genus level, these communities were mostly dominated by *Microcella* (44.9%), *Polynucleobacter* (28.3%), *Sphingomonas* (8.6%), *Thiobacter* (4.2%), *Legionella* (4%), *Methylobacterium* (1.2%), *Enterobacter* (0.9%), *Acinetobacter* (0.7%), *Paracoccus* (0.6%), and *Ilumatobacter* (0.5%). 

Skin microbiota from freshly dead fish (< 8h after death) was numerically dominated by the same triumvirate: *Proteobacteria* (76.1%), *Actinobacteria* (18.9%), and *Bacteroidetes* (4.6%) (Figure 3d). The 10 most abundant genera were *Acinetobacter* (16.4%), *Microcella* (14.1%), *Aeromonas* (11.4%), *Psychrobacter* (7.3%), *Citrobacter* (6.3%), *Obesumbacterium* (4.6%), *Polynucleobacter* (4.1%), *Paracoccus* (3.6%), *Propionibacterium* (3.4%), and *Sphingobacterium* (3.3%). Stressed fish microbiotas showed the least diverse taxonomic composition with a non-parametric Shannon index of 1.8 ± 0.6, followed by microbiotas from healthy fish (2.1 ± 1), and dead fish (2.6 ± 0.5) (Table S1). Differences in diversity were significant between stressed fish and dead fish only (t-test, p < 0.01).

### Taxonomic composition of environment

In order to highlight the origin of the fish skin microbiota, 30 bacterial consortium samples from surrounding environment were sequenced: water (n = 10), tank wall biofilm (n = 10), and biofilters (n = 10). Communities from biofilms, biofilters, and water samples were composed mostly by three major phyla: *Proteobacteria* (respectively 96.8%, 93.1%, 81.8%), *Actinobacteria* (respectively 0.5%, 2.1%, 16.8%) and *Bacteroidetes* (respectively 2.2%, 4.2%, 0.9%)(Figure 3c, 3e, 3f). 

At the genus level, water communities were mainly composed of *Polynucleobacter* (43.4%), *Microcella* (16.3%), *Legionella* (15.1%), *Azomonas* (6.6%), *Sphingomonas* (3.9%), *Novoshingobium* (1.7%), *Erythromicrobium* (1.5%), *Rhodoferax* (1.4%), *Duganella* (1.1%), and *Janthinobacterium* (0.9%). Tank biofilm contained principally *Bosea* (24.5%), *Sphingopyxis* (12.7%), *Devosia* (8.8%), *Legionella* (6.9%), *Sphingomonas* (5.3%), *Rhodobacter* (5.1%), *Novosphingobium* (4.9%), *Dinoroseobacter* (4.2%), and *Erythromicrobium* (2.8%). Biofilters were predominantly represented by *Nitrobacter* (40.3%), *Sphingopyxis* (10.7%), *Novosphingobium* (9.8%), *Erythromicrobium* (3.2%), *Kiloniella* (2.7%), *Anaeromyxobacter* (2.6%), *Dinoroseobacter* (1.9%), *Microcella* (1.8%), *Devosia* (1.6%), and *Sphingomonas* (1.8%).

Water bacterial consortia showed the least diverse taxonomic composition, harboring a non-parametric Shannon index of 1.6 ± 09, followed by biofilter (2.2 ± 0.2), and biofilm (2.6 ± 0.6) communities. These differences in diversity of the abundant bacterial communities were significant between water and biofilm (t-test, p < 0.05).

### Relationship between healthy fish skin mucus and environmental communities

**Figure 4:**
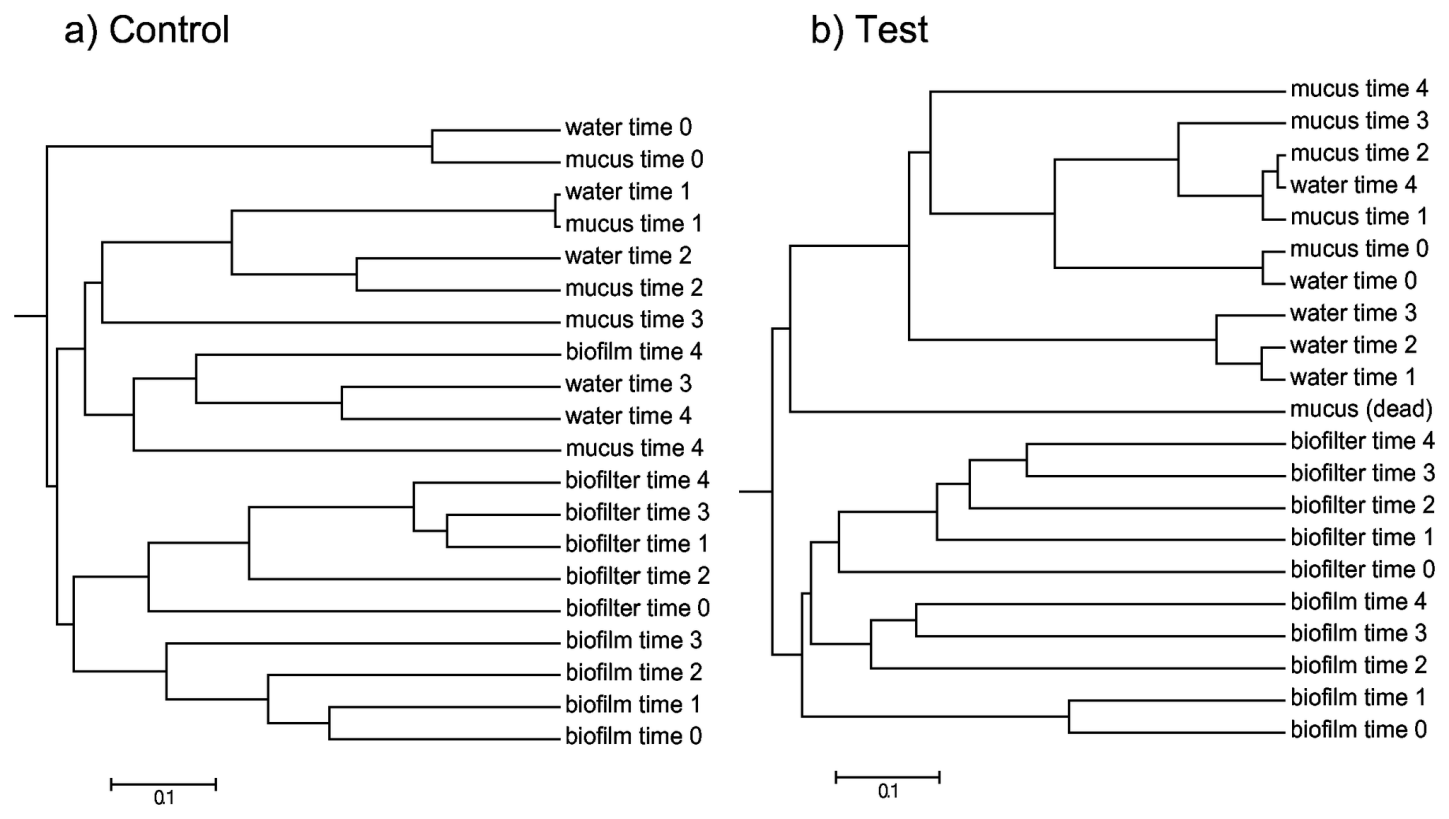
Theta index based dendrogram of bacterial communities from different samples. a) Samples from the control unit. Samples are represented by their sampling time (times 0 to 4). b) Samples from the test unit. Samples are represented by their sampling time (times 0 to 4). Sampling time: 1) before the stress trial (time 0), 2) stress trial at one week, 3) one week (time 1), 4) two weeks (time 2), and 5) three weeks (time 3), and 4 weeks after exposure to stress (time 4). Mucus bacterial communities from dead fish are also represented on this dendrogram.

In order to further characterize the relationships between water and fish skin mucus communities, we used a phylogenetic approach. We calculated the distance between communities taking into account the abundance of each OTU. The clustering of the different bacterial communities can be visualized by a dendrogram using the weighted Theta index of Yue and Clayton (Figure 4). The mucus samples from control group (Figure 4a) were closely related and mucus communities were closely related to the water bacterial community especially for the initial sampling times (times 0, 1 and 2), thus suggesting a temporal fluctuation for both water and mucus communities. The bacterial communities from bio-filter and biofilm clustered in the external branch of the dendrogram, indicating their high differentiation with mucosal and water communities. The second dendrogram showed that bacterial community from the stressed group (times 1, 2, 3 and 4) were no more closely related to the water community, whereas the initial sampling of mucus (time 0), which came from unstressed fish, closely clustered with water. The samples taken from dead fish exhibited a totally different microbiota, which clustered as an external branch (Figure 4b). Furthermore, to compare mucus bacterial community and water community, a Unifrac test was done by binning all of the sequences from water samples together and mucus samples together. The mean Unifrac distance was 0.26 and the p-value was lower than 0.001 indicating that those two bacterial communities are significantly different despite the close relationship observed in the dendrogram.

### Interactions between genera in bacterial communities of fish

**Figure 5:**
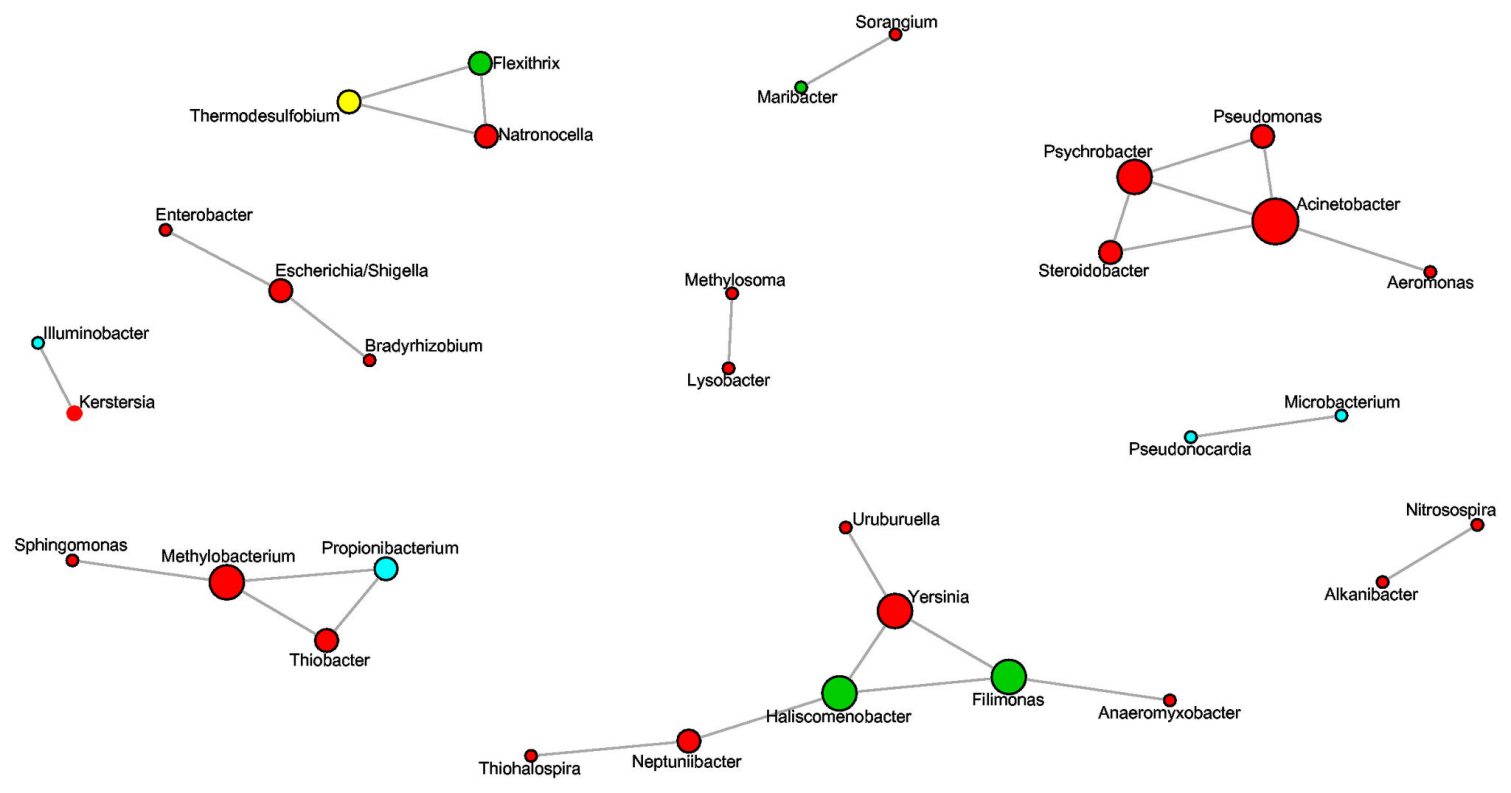
Network of co-occurring genera based on correlation analysis. A connection stands for a strong (Spearman’s r > 0.6) and significant (P-value < 0.01) correlation between two nodes (genera). The size of each node is proportional to the number of connections (i.e. degree). Colors indicate the phylum: red: *Proteobacteria*; blue: *Actinobacteria*; yellow: *Firmicutes*; green: *Bacteroidetes*.

We observed an overall non-random co-occurrence patterns in the whole dataset (C-score = 72.8, p = 0.01), which were then explored by a network inference based on Spearman’s correlations. Correlations were validated by selecting strong and highly significant correlations (σ > 0.6, p < 0.001). The resulting network contained 32 nodes and 26 edges (Figure 5). Ten groups of interactive genera composed from two to seven nodes were observed. Each node represented a genus, and some genera were specific to stressed and dead fish (*Psychrobacter*, *Steroidobacter*, *Pseudomonas*, *Acinetobacter*, *Aeromonas*) whereas others genera were mostly abundant on healthy fish (*Sphingomonas*, *Methylobacterium*, *Propionibacterium*, and *Thiobacter*). 

**Figure 6:**
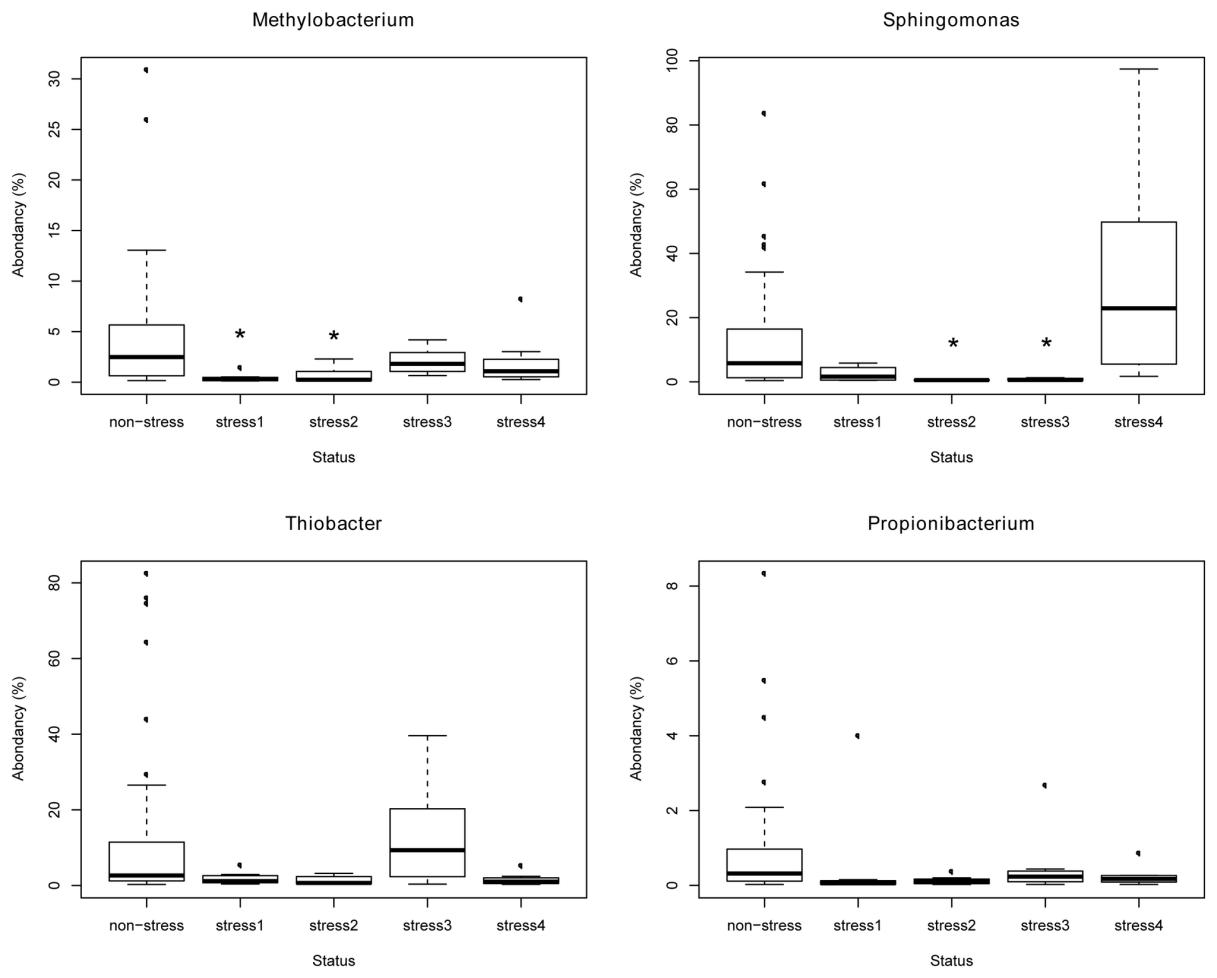
Evolution of relative abundance of beneficial genus from skin fish mucus following a stress event. Non-stress: sampling before stress; stress1: sampling 1 week after stress event; stress2: sampling 2 weeks after stress event; stress3: sampling 3 weeks after stress event; stress4: sampling 4 weeks after stress event. Statistical significant differences were tested by a Wilcoxon rank pair-test (p < 0.001).

**Figure 7:**
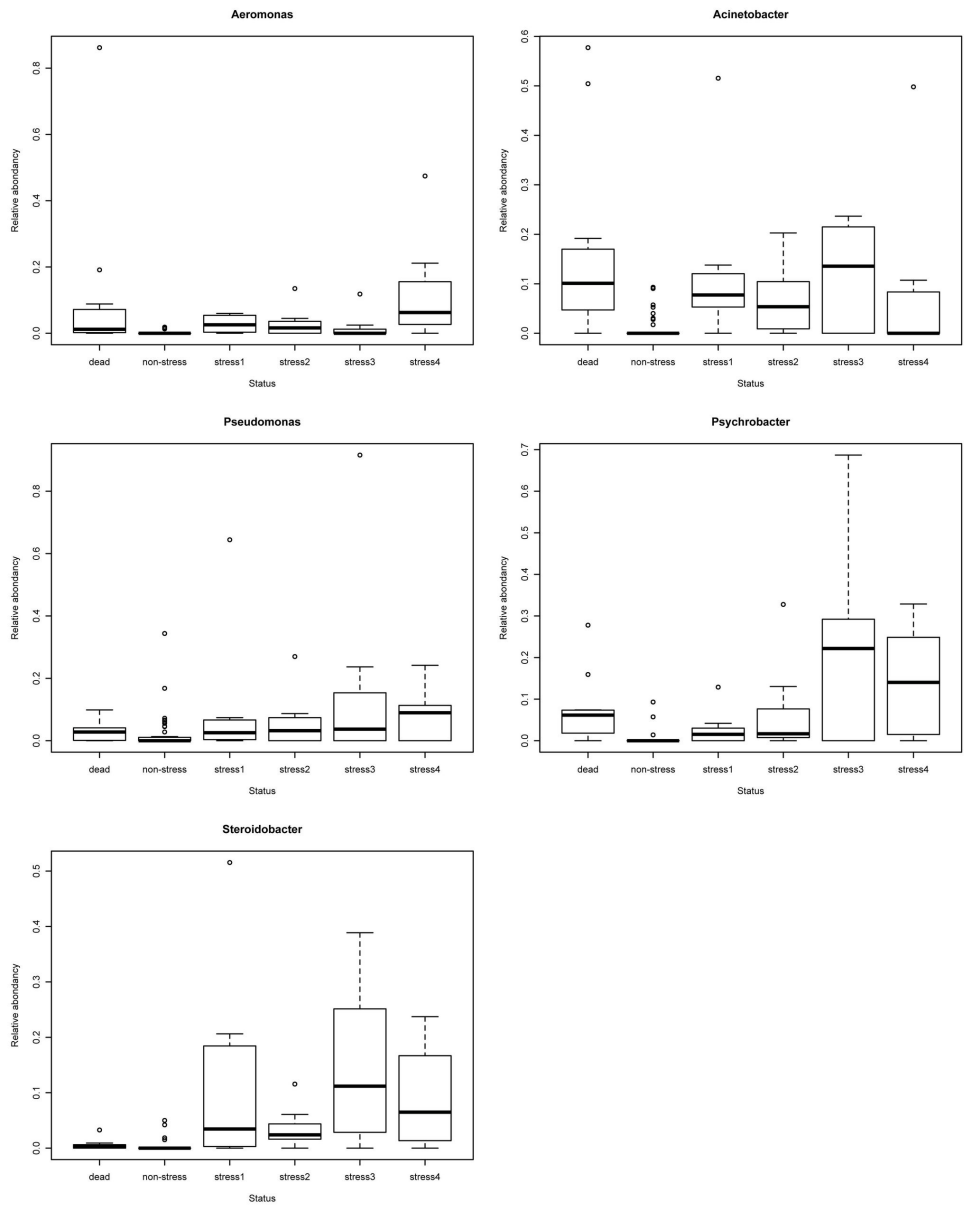
Evolution of relative abundance of beneficial genus from skin fish mucus following a stress event. Dead: sampling from Dead fish, Non-stress: sampling before stress; stress1: sampling 1 week after stress event; stress2: sampling 2 week after stress event; stress3: sampling 3 week after stress event; stress4: sampling 4 week after stress event. Statistical significant differences (represented by an asterisk) were tested by a Wilcoxon rank pair-test (p < 0.001).

The effect of stress trial was further explored by focusing on two consortia found in the network analysis (Figure 5). The consortium composed of *Sphingomonas*, *Methylobacterium*, *Propionibacterium*, and *Thiobacter* showed the same pattern of abundance variation during the experiment: a decrease occurred during the first two weeks following the stress trial followed by a recovery phase towards abundances as those measured before the stress trial (Table 1; Figure 6). In contrast, the consortium of bacteria which were mostly found on dead and stressed fish (*Psychrobacter*, *Steroidobacter*, *Pseudomonas*, *Acinetobacter*, *Aeromonas*) showed an important increase in abundance in fish mucus following stress exposure without further return to initial states (Table 2; Figure 7).

## Discussion

Our results suggest that brook charr skin microbiota is dynamic and is closely related to the water bacterial community. This is supported by the dendrogram cluster and also by the taxonomic classification at the genus level. In a global scale, water communities and healthy fish skin microbiota shared common genera (n = 110, i.e 72.8% of the whole community), which is in accordance with the hypothesis that skin microbiota results from the colonization of strains inhabiting the surrounding water [3]. However, Unifrac score calculated among samples showed that the structure of the bacterial communities from water and mucus were highly different (Unifrac test significance : p < 0.001), thus suggesting that fish skin mucus microbiota is highly specific. The distinction between those two types of samples (water and mucus) supports the hypothesis that each bacterial community is adapted sensu [36] to its own environment. Community structure changes to respond to a given factor (e.g. acute stress). These changes are the net effect of individual members’ successful and unsuccessful acclimation to the given factor. This shift in equilibrium is termed community adaptation.

The surrounding environment is not the only factor influencing brook charr skin microbiota. Thus, ANOVA results indicate that bacterial community of fish skin was sensitive to other factors; the stress of the host being the most influential. Indeed, our results showed that stressed fishes exhibited a different bacterial community when compared to non-stressed fishes and stressed fish communities were not closely related to the water community. Stressful conditions are known to modify the host skin mucus protein pattern [37] and could influence its immune response [22,38,39]. Such changes may alter the control of host on its bacterial community and disfavor the beneficial bacteria which stimulate the immune response and the production of inhibitory compounds [2,10,37,40]. Stress is known to trigger a shift in mucus protein composition, which in turn probably reshapes mucus microbial communities in favor of infectious pathogens [6,22,37]. An increase in plasma cortisol may have many effects on skin dermis and epidermis by inducing apoptosis of mucous cells [41]. The observed increase of *Steroidobacter* may benefit from steroidal hormone release as this is one of its source of nutrients [42]. 

The variation in abundance of numerous genera was also under the influence of the family genetic background. Furthermore, for 12 genera, the interaction between stress and family genetic background was statistically significant, suggesting that these two factors were not independent. To this respect, a previous study on brook charr physiological stress response showed that different strains of brook charr exhibited differential stress susceptibility [23]. Furthermore, Quantitative Traits Loci (QTL) of stress response were also identified in this species thus providing further support for a partial genetic basis of stress response [23,43].

Overall, skin mucus microbiota was widely dominated by *Proteobacteria*, especially *Polynucleobacter*, *Sphingomonas*, and *Thiobacter* genera. *Polynucleobacter* is an abundant genus in freshwater and is in some cases the most abundant [44]. *Actinobacteria* and *Bacteroidetes* were mostly represented by only one genus, respectively *Microcella* and *Flavobacterium*, both known to live in water and/or fish skin surface. Competition between indigenous bacteria and invading bacteria is also supported by our data. Indeed, bacterial communities from mucus formed complex interacting networks between genera. The results highlighted 27 co-occurrences between genera indicating that complementarities are present between those genera and/or that these genera share common functions (i.e. involving functional interdependency and/or sensitivity to the same environmental variations). Specific fish skin genera mainly co-occured in two strikingly contrasted clusters. The first one contained four genera: *Sphingomonas*, *Methylobacterium*, *Propionibacterium*, and *Thiobacter*. These genera were most abundant in the mucus of unstressed fish. Three of those genera are known to include species that have competitive properties which can explain their abundances in the natural microbiome of fish. *Methylobacterium* is the core of this network, and this genus is known to produce poly-β-hydroxybutyrate, which degrades short-chain fatty acid, and is known to inhibit the growth of pathogens [45,46]. *Sphingomonas paucimobilis* is known to inhibit the growth of fungi in plants [47]. *Propionibacterium* is known to synthesize bacteriocin, and has multiple beneficial effects on the host as enhancing health, stimulating immune response and increasing growth of other probiotic bacteria (*Bifidobacterium*). Though its capability of adhesion is very low, adhesion of other bacteria increases its own adhesion efficiency [48–50]. These four genera could hypothetically belong to a consortium in which interspecific interactions increase their respective fitness by synergy but also increase the host fitness. In such a case, they would be mutualists for each other but also for the host. The second cluster contains five genera (*Aeromonas*, *Psychrobacter*, *Pseudomonas*, *Acinetobacter, Steroidobacter*) among which four exhibited well documented pathogenic properties [4]. Indeed, these genera contained four species (*Aeromonas salmonicida*, *Acinetobacter* *spp*, *Pseudomonas chlororaphis, Psychrobacter immobilis*) that are considered as opportunistic pathogens in salmonids [4,51]. 

In conclusion, this study demonstrates that physiological stress may induce changes in fish microbiota, whereby beneficial bacteria decrease following a stress event, thus resulting in an empty niche for opportunistic pathogens. This result highlights the importance of mutualistic bacterial function as front-line protection for the host. Furthermore, our results indicate that microbiota dysbiosis is one of the conditions which triggers opportunistic infections. These observations pave the way to the development of a new alternative strategy to prevent opportunistic infections by using probiotics. Probiotics can maintain homeostasis by direct competition with pathogens [52]. In the context of aquaculture, one efficient approach to prevent and treat opportunistic infections would be to establish a probiotic treatment regime from the beneficial bacteria isolated from host microbiota that exhibited clear antagonistic effects against pathogens [53,54]. Finally, we showed that bacteria interact as taxonomic networks in fish skin. Investigating the bacterial interactions could be further explored using a metatranscriptomic approach. This would provide insight towards elucidating the functional interactions between each actor of the network, as well as characterizing the mechanisms underlying the inter-dependence between host and bacteria. 

## Supporting Information

Table S1 **Sampling description and sequencing data for the abundant biosphere.** (Nseqs: numbers of high quality reads; coverage: Good’s coverage estimation (%); npshannon: non-parametric Shannon index).
(DOC)
