# Supplementary material for: Network Analysis Highlights Complex Interactions between Pathogen, Host and Commensal Microbiota
Source: PLoS One. 2013 Dec 23;8(12):e84772. doi: 10.1371/journal.pone.0084772 (PMC3871659; doi:10.1371/journal.pone.0084772)
Supplement: Table S1 — Sampling description and sequencing data for the abundant biosphere. (Nseqs: numbers of high quality reads; coverage: Good’s coverage estimation (%); npshannon: non-parametric Shannon index). (DOC) [file pone.0084772.s001.doc]

Supplementary table 1: Sampling description and sequencing data for the abundant biosphere. (Nseqs: numbers of high quality reads; coverage: Good’s coverage estimations (%); npshannon: non-parametric Shannon index).

| **Sample** | **Environment** | **nseqs** | **coverage** | **npshannon** |
| --- | --- | --- | --- | --- |
| **NBFMT1** | Biofilm | 62 | 0.919355 | 1.521151 |
| **NBFMT2** | Biofilm | 1272 | 0.98978 | 1.474674 |
| **NBFMT3** | Biofilm | 616 | 0.982143 | 2.844787 |
| **NBFMT4** | Biofilm | 44 | 0.704545 | 3.259696 |
| **NBFMT5** | Biofilm | 50 | 0.86 | 2.775451 |
| **SBFMT1** | Biofilm | 170 | 0.964706 | 2.547486 |
| **SBFMT2** | Biofilm | 855 | 0.980117 | 2.995345 |
| **SBFMT3** | Biofilm | 160 | 0.8625 | 3.183568 |
| **SBFMT4** | Biofilm | 112 | 0.910714 | 2.67309 |
| **SBFMT5** | Biofilm | 127 | 0.905512 | 3.12859 |
| **NBFTT1** | Biofilter | 1831 | 0.993446 | 0.479365 |
| **NBFTT2** | Biofilter | 593 | 0.971332 | 1.923485 |
| **NBFTT3** | Biofilter | 123 | 0.886179 | 2.568913 |
| **NBFTT4** | Biofilter | 99 | 0.949495 | 0.801856 |
| **NBFTT5** | Biofilter | 128 | 0.898438 | 2.029436 |
| **SBFTT1** | Biofilter | 216 | 0.930556 | 2.157271 |
| **SBFTT2** | Biofilter | 922 | 0.954447 | 3.655179 |
| **SBFTT3** | Biofilter | 58 | 0.827586 | 2.53595 |
| **SBFTT4** | Biofilter | 111 | 0.801802 | 3.365083 |
| **SBFTT5** | Biofilter | 81 | 0.82716 | 2.96179 |
| **NB1CT1** | Non-stress Skin Fish Mucus | 1888 | 0.992585 | 2.748277 |
| **NB1CT2** | Non-stress Skin Fish Mucus | 8826 | 0.995921 | 0.562001 |
| **NB1CT3** | Non-stress Skin Fish Mucus | 1529 | 0.976455 | 2.077291 |
| **NB1CT4** | Non-stress Skin Fish Mucus | 1895 | 0.984697 | 2.027658 |
| **NB1CT5** | Non-stress Skin Fish Mucus | 414 | 0.942029 | 2.845815 |
| **NB1ST1** | Non-stress Skin Fish Mucus | 1648 | 0.989078 | 3.333587 |
| **NB1ST2** | Non-stress Skin Fish Mucus | 11272 | 0.996895 | 0.635817 |
| **NB1ST3** | Non-stress Skin Fish Mucus | 3301 | 0.990912 | 2.213552 |
| **NB1ST4** | Non-stress Skin Fish Mucus | 4332 | 0.996076 | 1.090961 |
| **NB1ST5** | Non-stress Skin Fish Mucus | 998 | 0.970942 | 2.422787 |
| **NB2CT1** | Non-stress Skin Fish Mucus | 1389 | 0.989201 | 3.596767 |
| **NB2CT2** | Non-stress Skin Fish Mucus | 2272 | 0.984595 | 1.017274 |
| **NB2CT3** | Non-stress Skin Fish Mucus | 1162 | 0.975904 | 2.502896 |
| **NB2CT4** | Non-stress Skin Fish Mucus | 2449 | 0.988975 | 1.504601 |
| **NB2CT5** | Non-stress Skin Fish Mucus | 1058 | 0.975425 | 1.802685 |
| **NB2ST1** | Non-stress Skin Fish Mucus | 1206 | 0.978441 | 3.486359 |
| **NB2ST2** | Non-stress Skin Fish Mucus | 7199 | 0.996805 | 1.003661 |
| **NB2ST3** | Non-stress Skin Fish Mucus | 590 | 0.964407 | 2.180598 |
| **NB2ST4** | Non-stress Skin Fish Mucus | 1232 | 0.974026 | 2.918966 |
| **NB2ST5** | Non-stress Skin Fish Mucus | 68 | 0.794118 | 2.858702 |
| **NB3CT1** | Non-stress Skin Fish Mucus | 363 | 0.917355 | 3.69571 |
| **NB3CT2** | Non-stress Skin Fish Mucus | 10227 | 0.995698 | 0.482661 |
| **NB3CT3** | Non-stress Skin Fish Mucus | 1682 | 0.988109 | 2.868609 |
| **NB3CT4** | Non-stress Skin Fish Mucus | 636 | 0.935535 | 3.189344 |
| **NB3CT5** | Non-stress Skin Fish Mucus | 702 | 0.961538 | 2.565408 |
| **NB3ST1** | Non-stress Skin Fish Mucus | 291 | 0.893471 | 3.871104 |
| **NB3ST2** | Non-stress Skin Fish Mucus | 2073 | 0.988423 | 0.557806 |
| **NB3ST3** | Non-stress Skin Fish Mucus | 1526 | 0.973788 | 2.569386 |
| **NB3ST4** | Non-stress Skin Fish Mucus | 703 | 0.975818 | 2.921896 |
| **NB3ST5** | Non-stress Skin Fish Mucus | 323 | 0.888545 | 3.622939 |
| **NB4CT1** | Non-stress Skin Fish Mucus | 348 | 0.942529 | 3.227155 |
| **NB4CT2** | Non-stress Skin Fish Mucus | 10657 | 0.997373 | 0.347214 |
| **NB4CT3** | Non-stress Skin Fish Mucus | 2976 | 0.995632 | 2.273233 |
| **NB4CT4** | Non-stress Skin Fish Mucus | 1452 | 0.985537 | 1.715325 |
| **NB4CT5** | Non-stress Skin Fish Mucus | 428 | 0.96729 | 3.082689 |
| **NB4ST1** | Non-stress Skin Fish Mucus | 1294 | 0.983771 | 2.848421 |
| **NB4ST2** | Non-stress Skin Fish Mucus | 1086 | 0.982505 | 0.839126 |
| **NB4ST3** | Non-stress Skin Fish Mucus | 1518 | 0.979578 | 2.658584 |
| **NB4ST4** | Non-stress Skin Fish Mucus | 593 | 0.973019 | 2.615002 |
| **NB4ST5** | Non-stress Skin Fish Mucus | 986 | 0.978702 | 2.300629 |
| **SB1CT1** | Non-stress Skin Fish Mucus | 1745 | 0.986819 | 1.326118 |
| **SB1ST1** | Non-stress Skin Fish Mucus | 2211 | 0.987788 | 0.904489 |
| **SB2CT1** | Non-stress Skin Fish Mucus | 17345 | 0.997982 | 0.976691 |
| **SB2ST1** | Non-stress Skin Fish Mucus | 7608 | 0.995925 | 1.000238 |
| **SB3CT1** | Non-stress Skin Fish Mucus | 9993 | 0.996397 | 0.888202 |
| **SB3ST1** | Non-stress Skin Fish Mucus | 6585 | 0.994381 | 0.959124 |
| **SB4CT1** | Non-stress Skin Fish Mucus | 7135 | 0.994394 | 1.126947 |
| **SB4ST1** | Non-stress Skin Fish Mucus | 5374 | 0.990882 | 1.233004 |
| **SB1SDEAD12** | Skin Dead Fish Mucus | 243 | 0.975309 | 2.380039 |
| **SB1SDEAD13** | Skin Dead Fish Mucus | 427 | 0.971897 | 3.130885 |
| **SB1SDEAD14** | Skin Dead Fish Mucus | 529 | 0.971645 | 2.620063 |
| **SB2CDEAD10** | Skin Dead Fish Mucus | 439 | 0.986333 | 2.234227 |
| **SB2CDEAD2** | Skin Dead Fish Mucus | 609 | 0.968801 | 3.150274 |
| **SB2CDEAD4** | Skin Dead Fish Mucus | 438 | 0.972603 | 2.709864 |
| **SB2CDEAD5** | Skin Dead Fish Mucus | 6313 | 0.997307 | 1.334191 |
| **SB2CDEAD6** | Skin Dead Fish Mucus | 543 | 0.965009 | 2.860729 |
| **SB2SDEAD11** | Skin Dead Fish Mucus | 115 | 0.965217 | 2.251103 |
| **SB3SDEAD1** | Skin Dead Fish Mucus | 343 | 0.953353 | 2.617072 |
| **SB3SDEAD7** | Skin Dead Fish Mucus | 584 | 0.94863 | 3.299458 |
| **SB1CT2** | Stressed Skin Fish Mucus | 776 | 0.962629 | 2.871249 |
| **SB1CT3** | Stressed Skin Fish Mucus | 6441 | 0.991306 | 1.241758 |
| **SB1CT4** | Stressed Skin Fish Mucus | 897 | 0.976589 | 1.790966 |
| **SB1CT5** | Stressed Skin Fish Mucus | 3042 | 0.988494 | 2.087144 |
| **SB1ST2** | Stressed Skin Fish Mucus | 6763 | 0.994677 | 1.910522 |
| **SB1ST3** | Stressed Skin Fish Mucus | 6905 | 0.993483 | 1.320484 |
| **SB1ST4** | Stressed Skin Fish Mucus | 2030 | 0.982759 | 2.816844 |
| **SB1ST5** | Stressed Skin Fish Mucus | 1677 | 0.975552 | 2.035152 |
| **SB2CT2** | Stressed Skin Fish Mucus | 1762 | 0.986947 | 1.508734 |
| **SB2CT3** | Stressed Skin Fish Mucus | 4440 | 0.988964 | 1.243259 |
| **SB2CT4** | Stressed Skin Fish Mucus | 4054 | 0.983473 | 2.105006 |
| **SB2CT5** | Stressed Skin Fish Mucus | 3309 | 0.987912 | 2.241472 |
| **SB2ST2** | Stressed Skin Fish Mucus | 3878 | 0.993038 | 1.741418 |
| **SB2ST3** | Stressed Skin Fish Mucus | 11233 | 0.994481 | 1.156511 |
| **SB2ST4** | Stressed Skin Fish Mucus | 973 | 0.979445 | 2.976453 |
| **SB2ST5** | Stressed Skin Fish Mucus | 1865 | 0.983378 | 2.313899 |
| **SB3CT2** | Stressed Skin Fish Mucus | 16216 | 0.996238 | 1.279789 |
| **SB3CT3** | Stressed Skin Fish Mucus | 3936 | 0.992124 | 1.32301 |
| **SB3CT4** | Stressed Skin Fish Mucus | 1747 | 0.972524 | 2.777932 |
| **SB3CT5** | Stressed Skin Fish Mucus | 1462 | 0.991792 | 0.839555 |
| **SB3ST2** | Stressed Skin Fish Mucus | 18340 | 0.995911 | 1.114482 |
| **SB3ST3** | Stressed Skin Fish Mucus | 5188 | 0.993446 | 1.925913 |
| **SB3ST4** | Stressed Skin Fish Mucus | 995 | 0.949749 | 2.067678 |
| **SB3ST5** | Stressed Skin Fish Mucus | 843 | 0.960854 | 2.167543 |
| **SB4CT2** | Stressed Skin Fish Mucus | 16693 | 0.996106 | 1.249698 |
| **SB4CT3** | Stressed Skin Fish Mucus | 6579 | 0.995136 | 1.604749 |
| **SB4CT4** | Stressed Skin Fish Mucus | 2829 | 0.985861 | 2.1413 |
| **SB4CT5** | Stressed Skin Fish Mucus | 3706 | 0.991905 | 1.817797 |
| **SB4ST2** | Stressed Skin Fish Mucus | 14019 | 0.995863 | 1.204816 |
| **SB4ST3** | Stressed Skin Fish Mucus | 5574 | 0.992106 | 1.495252 |
| **SB4ST4** | Stressed Skin Fish Mucus | 1690 | 0.97929 | 2.077642 |
| **SB4ST5** | Stressed Skin Fish Mucus | 1004 | 0.961155 | 2.055942 |
| **NEAUT1** | Water | 3024 | 0.994709 | 1.878797 |
| **NEAUT2** | Water | 1399 | 0.996426 | 0.249401 |
| **NEAUT3** | Water | 1892 | 0.989429 | 1.132107 |
| **NEAUT4** | Water | 1031 | 0.971872 | 3.205031 |
| **NEAUT5** | Water | 359 | 0.922006 | 3.1557 |
| **SEAUT1** | Water | 10280 | 0.997374 | 1.35943 |
| **SEAUT2** | Water | 6431 | 0.994558 | 0.701569 |
| **SEAUT3** | Water | 6509 | 0.995237 | 1.153778 |
| **SEAUT4** | Water | 4417 | 0.990491 | 1.725993 |
| **SEAUT5** | Water | 9190 | 0.99445 | 1.59311 |
